# Growth and disease burden in children with hypophosphatasia

**DOI:** 10.1530/EC-22-0240

**Published:** 2023-04-25

**Authors:** Wolfgang Högler, Agnès Linglart, Anna Petryk, Priya S Kishnani, Lothar Seefried, Shona Fang, Cheryl Rockman-Greenberg, Keiichi Ozono, Kathryn Dahir, Gabriel Ángel Martos-Moreno

**Affiliations:** 1 Department of Paediatrics and Adolescent Medicine, Johannes Kepler University Linz, Linz, Austria; 2 Institute of Metabolism and Systems Research, University of Birmingham, Birmingham, UK; 3 AP-HP, Hôpital Bicêtre Paris Saclay, service d’endocrinologie et diabète de l’enfant, DMU 3 SEA, centre de référence des maladies rares du métabolisme du calcium et du phosphate, filière OSCAR; Université de Paris-Saclay INSERM U1185, Hôpital Bicêtre, Le Kremlin-Bicêtre, France; 4 Alexion, AstraZeneca Rare Disease, Boston, Massachusetts, USA; 5 Duke University Medical Center, Durham, North Carolina, USA; 6 University of Würzburg, Würzburg, Germany; 7 University of Manitoba, Winnipeg, Manitoba, Canada; 8 Osaka University, Suita, Osaka, Japan; 9 Vanderbilt University Medical Center, Nashville, Tennessee, USA; 10 Departments of Pediatrics and Pediatric Endocrinology Hospital Infantil Universitario Niño Jesús, IIS La Princesa, Universidad Autónoma de Madrid, CIBERobn, ISCIII, Madrid, Spain

**Keywords:** height, body mass index, registry, hypophosphatasia, growth

## Abstract

**Objective:**

Hypophosphatasia, an inborn error of metabolism characterized by impaired bone mineralization, can affect growth. This study evaluated relationships between anthropometric parameters (height, weight, and body mass index) and clinical manifestations of hypophosphatasia in children.

**Design:**

Data from children (aged <18 years) with hypophosphatasia were analyzed from the observational Global Hypophosphatasia Registry.

**Methods:**

Anthropometric parameters were evaluated by age group (<2 years and ≥2 years) at assessment. The frequency of hypophosphatasia manifestations was compared between children with short stature (< percentile) and those with normal stature.

**Results:**

This analysis included 215 children (54.4% girls). Short stature presented in 16.1% of children aged <2 years and 20.4% of those aged ≥2 years at assessment. Among those with available data (*n* = 62), height was below the target height (mean: −0.66 standard deviations). Substantial worsening of growth (mean delta height *z* score: −1.45; delta weight *z* score: −0.68) occurred before 2 years of age, while in those aged ≥2 years, anthropometric trajectories were maintained (delta height *z* score: 0.08; delta weight *z* score: 0.13). Broad-ranging hypophosphatasia manifestations (beyond dental) were observed in most children.

**Conclusions:**

Short stature was not a consistent characteristic of children with hypophosphatasia, but growth impairment was observed in those aged <2 years, indicating that hypophosphatasia might affect growth plate activity during infancy. In addition, a broad range of clinical manifestations occurred in those above and below the third percentile for height, suggesting that height alone may not accurately reflect hypophosphatasia disease burden and that weight is less affected than longitudinal growth.

## Introduction

Hypophosphatasia (HPP) is a rare, inherited, metabolic disorder caused by impaired bone mineralization due to tissue nonspecific alkaline phosphatase deficiency (1). HPP can manifest throughout life (*in utero* to adulthood) with skeletal and nonskeletal symptoms that may negatively affect growth (2, 3). A natural history study of longitudinal data collected from 101 patients over the course of 25 years at a single center in the United States showed that children with HPP may have below-average height but normal weight when compared with the general population (3).

Recent evidence from the Global HPP Registry, a multinational, longitudinal study of patients with HPP, showed that bone deformity, failure to thrive, and rickets-like changes were some of the most commonly reported manifestations of HPP in children (4). Given that hypomineralization disorders can negatively affect the zone of hypertrophic chondrocytes of the growth plate, growth in children with HPP has often been suggested as an indicator of disease burden and overall health (5, 6, 7). This analysis aimed to explore the anthropometric characteristics of children with HPP using real-world data collected during routine clinical practice and to study the relationship between height impairment and other clinical manifestations of HPP to better understand the disease burden.

## Methods

### Data source and informed consent

The Global HPP Registry is an observational, prospective, multinational study (NCT02306720; EUPAS13514) of patients with HPP (4, 8). The registry was initiated in 2015 with the purpose of collecting longitudinal data, including patient demographics and clinical characteristics of HPP, such as age at first HPP manifestation, family history of HPP, signs and symptoms, height, and weight, from consenting and assenting patients with HPP (or their parents or guardians as appropriate) (4, 8). All data were collected during routine clinical visits (8). Data recorded up to December 7, 2020, were included in this analysis.

As previously described, all aspects of the Global HPP Registry are sponsored by Alexion, AstraZeneca Rare Disease (Boston, MA, USA), and the registry is monitored by a scientific advisory board with clinical expertise in HPP (4). The study protocol was approved by the institutional review board (or local equivalent) of participating study sites (see Acknowledgements section) and is being conducted in accordance with the European Medicines Agency Good pharmacovigilance practices, the International Society for Pharmacoepidemiology Guidelines for Good Pharmacoepidemiology Practices, and the World Medical Association Declaration of Helsinki. Before participating, all patients and/or their parents/legal guardians provided written informed consent and approval to release medical records.

### Patient population

Registry data from children (aged <18 years) with HPP were included in the study (4, 8). The diagnosis of HPP was determined by the investigating physician who enrolled the patient in the Registry. Further, enrolled patients were required to have documentation of low serum alkaline phosphatase (ALP) activity under the age- and sex-adjusted reference range and/or a genetic test indicating at least one *ALPL* variant (classified as either pathogenic, likely pathogenic, or variant of uncertain significance) within the Registry. All data used in this study were from prior to the start of enzyme replacement therapy (asfotase alfa). Eligible patients were required to have available data on registry enrollment date, date of birth or age at registry enrollment, sex, asfotase alfa treatment start date (if treated), and at least one height measurement before treatment initiation (if treated); treatment was not a requirement for inclusion and some patients in the Global HPP Registry are untreated. Eligible patients were also required to be full-term at birth (defined as ≥37 weeks of gestation). To avoid the bias of prematurity on anthropometric outcomes, patients born preterm were not included in the study.

### Anthropometric and clinical characteristics

**Figure 1:**
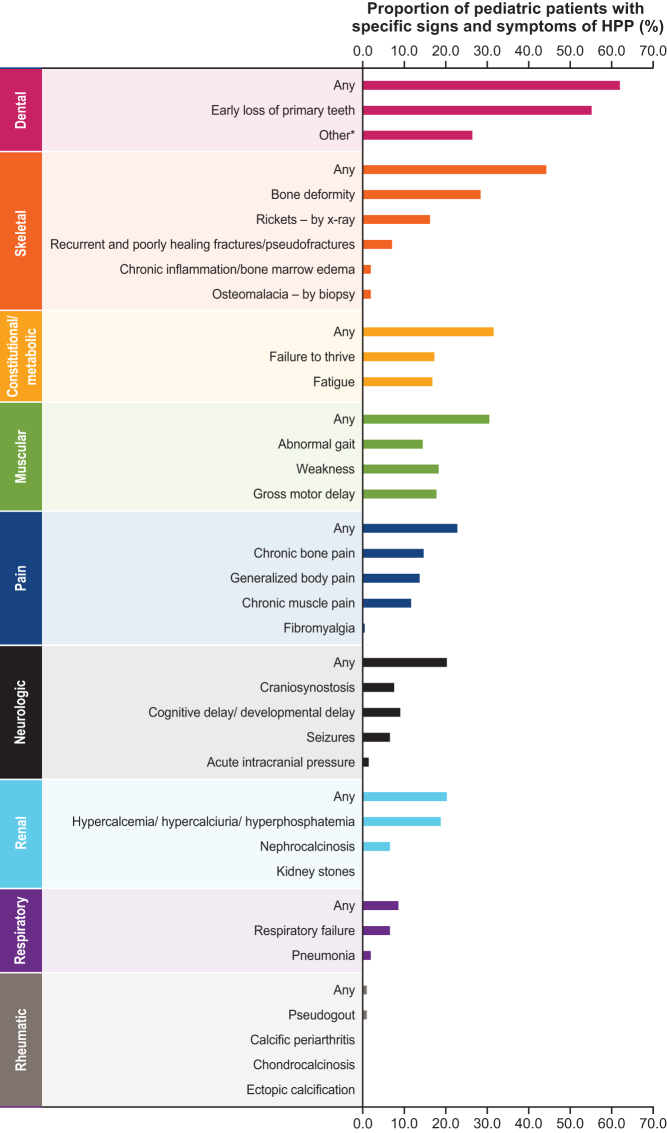
Frequency of specific signs and symptoms of HPP in children (*n* = 197). *Includes loss of permanent teeth, loose teeth, poor dentition, hypodontia, dental implants, dental bridges, and dentures. HPP, hypophosphatasia.

Baseline, cross-sectional characteristics of the study population that were analyzed included patient demographics, age at first HPP manifestation (age <6 months: perinatal/infantile-onset HPP; age 6 months to 18 years: juvenile-onset HPP), family history of HPP (yes, no, or unknown status), number of baseline signs and symptoms of HPP (based on those that are listed in Fig. 1), and low full-term birth weight (defined as <2.5 kg (9, 11)). For anthropometric outcomes, the first available height, weight, and body mass index (BMI) measurements were evaluated by age group (<2 and ≥2 years; note, height measurements in those <2 years are referred to as length). BMI in kg/m^2^ was derived from the recorded height and weight measurements. In line with Centers for Disease Control (CDC) recommendations, height, weight, and BMI *z* scores for age and sex were calculated using World Health Organization (WHO) standards for measurements obtained at age <2 years and CDC standards for measurements obtained at age ≥2 years (11, 13).

To evaluate longitudinal growth patterns, change in height, weight, and BMI *z* scores were calculated from the first available measurement to the last available measurement in those <2 and ≥2 years, separately. In addition, for each assessment age group (<2 and ≥2 years), subgroup analyses were conducted to evaluate the change in height, weight, and BMI *z* scores by age at first HPP manifestation (aged <6 months vs aged 6 months to <18 years at first HPP signs or symptoms); subgroup analyses were also conducted to assess catch-up growth in children with height measurements below the third percentile at first assessment. Those above and below the third percentile at the last recorded measurement were also compared with regard to the frequency of baseline signs and symptoms of HPP.

Target height *z* scores based on modified midparental height (MPH) were evaluated in children with assessments ≥2 years. MPH was determined using the Tanner method (13). The MPH was converted to a *z* score (MPH Z) using the CDC growth standards for 18-year-old males or females, as appropriate.

### Sensitivity analyses

Two sensitivity analyses were conducted to assess the robustness of the main results. First, birth length was excluded as a first height measurement when analyzing longitudinal growth (change in height over time) for those aged <2 years to account for the higher possibility of errors in birth length measurements and exclude possible artifacts due to the rapid growth in early infancy. A second sensitivity analysis was performed among the patients who had measurements in both age groups, to ensure that the findings in the main analysis were not biased by those who had measurements in only one age group.

### Statistical analysis

Implausible height and weight values (data entry errors) were identified by visual inspection of the individual growth plots and by examination of the summary statistics by age group. Implausible values were queried for correction or otherwise excluded from all analyses.

All analyses were descriptive. Continuous variables were described using median (min, max) and mean (s.d.), as appropriate. Categorical variables were described using frequencies and percentages.

A minimum duration of 2 months (for patients aged <2 years) and ≥6 months (for patients aged ≥2 years) was required between measurements for all analyses that investigated the change in anthropometric *z* scores. To assess growth relative to the target MPH, the difference between a child’s height *z* score at last assessment (HT Z) and the modified MPH Z was determined (i.e. delta HT Z – MPH Z). The delta HT Z – MPH Z was further evaluated by parental HPP status. Prevalence ratios comparing the frequency of signs and symptoms by height percentile were calculated as the ratio of the proportion of children below the third percentile for height vs children at or above the third percentile for height with a specific sign or symptom of interest; 95% confidence intervals were determined using the binomial approximation. All analyses were conducted separately based on age and sex. Statistical analyses were performed using SAS Life Science Analytics Framework 5.2.1 (Cary, NC, USA).

## Results

### Baseline characteristics of children with HPP

This analysis included data from 215 enzyme replacement therapy (asfotase alfa)-naïve children with HPP. The baseline clinical characteristics of the study population are shown in Table 1. Most children were female (54.4%) and experienced their first HPP manifestation after 6 months of age (59.5%). In addition, 26.5 and 16.7% had a record of at least one parent or other family members (e.g. sibling) diagnosed with HPP, respectively. Low birth weights (<2.5 kg) at full term occurred in 7.4% of the 215 children included in this study.

**Table 1:** Baseline characteristics of the study population.

Characteristic	Children with HPP (aged <18 years at baseline, *n* = 215)
Sex, *n* (%)	
Male	98 (45.6)
Female	117 (54.4)
Age group at first HPP manifestation, *n* (%)	
Aged <6 months	60 (27.9)
Aged 6 months to <18 years	128 (59.5)
Unknown	27 (12.6)
Low birth weight at full-term,^a^*n* (%)	
Yes	16 (7.4)
No	185 (86.0)
Unknown	14 (6.5)
Family history of HPP, *n* (%)	
Yes	93 (43.3)
No	112 (52.1)
Unknown	10 (4.7)
If yes,	
Parent(s)	57 (26.5)
Mother	40 (18.6)
Father	22 (10.2)
Both	5 (2.3)
Other^b^	36 (16.7)
Number of baseline signs/symptoms per patient	
Mean (s.d.)	3.2 (2.7)^c^
Median (min, max)	2 (1, 18)^c^
Number of baseline signs/symptoms per patient, *n* (%)	
1	65 (33.0)^c^
2	44 (22.3)^c^
3	25 (12.7)^c^
4	19 (9.6)^c^
5+	44 (22.3)^c^

^a^Low birth weight was defined as birth weight strictly <2.5 kg and the pregnancy was full-term (defined as ≥37 weeks of gestation).

^b^Includes other family members, such as siblings, grandparents, aunts, uncles, and cousins.

^c^*n* = 197.

HPP, hypophosphatasia; max, maximum; min, minimum.

### Children with HPP had a wide range of disease manifestations

Data on signs and symptoms of HPP were available for 197 children where a majority (67.0%) experienced ≥2 signs and symptoms of HPP with a median (min, max) of 2 (1, 18). When assessing the frequency of specific signs and symptoms of HPP, dental and skeletal manifestations were most common, followed by constitutional/metabolic, muscular, pain, neurological, and renal manifestations. Each of these manifestations was recorded in ≥20% of children (Fig. 1), indicating that children with HPP are broadly affected beyond the skeletal system. At least one key skeletal HPP manifestation was recorded in 79.2% of children, with bowing of the long bones of the legs and rickets being most common (Table 2).

**Table 2:** Key skeletal signs and symptoms of HPP in children.

	Children with data on HPP-related skeletal manifestations (*n* = 183)
Patients with key skeletal manifestations, *n* (%)	145 (79.2)
Number of manifestations	382
Bowing of long bones in legs	31 (21.4)
Rickets	27 (18.6)
Abnormally shaped chest	17 (11.7)
Bowing of long bones in arms	19 (13.1)
Abnormally shaped skull	13 (9.0)
Abnormal gait	13 (9.0)
Scoliosis	9 (6.2)
Club foot deformity	4 (2.8)
Osteomalacia – by biopsy	4 (2.8)
Bone pain severe enough to limit patient’s activities	3 (2.1)
Bone pain severe enough to require pain medication^a^	5 (3.4)

^a^Use of pain medication, such as type (opioid, analgesic, etc) or frequency, was not predefined and was reported at the discretion of the investigating physician.

HPP, hypophosphatasia.

### Most children with HPP did not present with short stature

**Figure 2:**
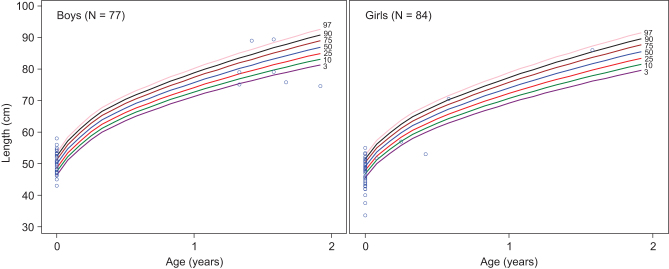
First length measurement in children with HPP aged <2 years. Length measurements were analyzed in 161 children aged <2 years and are shown according to the WHO standards for length. HPP, hypophosphatasia; WHO, World Health Organization.

**Figure 3:**
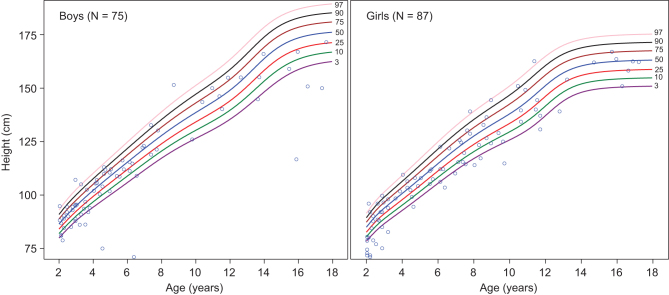
First height measurement in children with HPP aged ≥2 years. Height measurements were analyzed in 162 children aged ≥2 years and are shown according to the CDC standards for height. CDC, Centers for Disease Control and Prevention; HPP, hypophosphatasia.

To investigate the prevalence of short stature, data from the first available measurement of height were evaluated. Children were, on average, within the normal height range before 2 years of age (mean (s.d.) height *z* scores: −0.28 (2.27); *n* = 162), and after 2 years of age (−0.70 (1.75); *n* = 162). At the first available measurement, ≥50% of children were below the 50th percentile for height regardless of age (<2 years: 50.3%; ≥2 years: 61.7%). Individual heights were plotted on growth charts in Fig. 2 (<2 years) and Fig. 3 (≥2 years). Short stature (< third percentile) was observed in a subset of children, irrespective of the age group of the measurement (<2 years: 16.7%; ≥2 years: 20.4%). Most children were also within the normal weight range at <2 years (mean (s.d.) *z* score: −0.09 (1.31); *n* = 205) and ≥2 years of age (−0.53 (2.18); *n* = 160). Only 5.9% of children <2 years of age (Supplementary Fig. 1, see section on supplementary materials given at the end of this article) and 16.9% at ≥2 years of age (Supplementary Fig. 2) were below the third weight percentile in their respective age groups. Given that failure to thrive may be a sign of HPP in young children, we also quantified the proportion of children with low BMI (< third percentile), which was 8.1% in children <2 years of age (Supplementary Fig. 3) and 6.3% in children ≥2 years of age (Supplementary Fig. 4), which was more than in the normal population (expected 3%).

### Growth was impaired in the first 2 years of life but stabilized thereafter

Results of the longitudinal growth analysis based on the change from the first to last available measurement showed that children aged <2 years had impaired growth (mean (s.d .) delta length *z* score: −1.45 (1.89); *n* = 44) and impaired weight gain (mean (s.d.) delta weight *z* score: −0.68 (1.91); *n* = 49) over time, while children aged ≥2 years maintained their height percentiles (mean (s.d.) delta height *z* score: 0.08 (0.53); *n* = 102) and weight percentiles (mean (s.d .) delta weight *z* score: 0.13 (0.73); *n* = 101) over time (Table 3 and Supplementary Table 1). At the last available measurement, 64.5% of children were below the 50th percentile for height (<2 years: 89.6%; ≥2 years: 58.1%) and 23.2% below the third percentile (<2 years: 54.5%; ≥2 years: 19.6%). Thus, a substantial proportion of children under 2 years of age remained short in stature. BMI *z* scores did not appear to worsen over time in children aged <2 or ≥2 years (Supplementary Table 1).

**Table 3:** Changes in height^a^ over time (based on at least two consecutive measurements per patient) in children with HPP.

	Height of children with HPP aged <2 years at assessment	Height of children with HPP aged ≥2 years at assessment
First measurement *z* score, *n*	44	102
Mean (s.d.)	−0.58 (1.90)	−0.62 (1.54)
Median (min, Q1, Q3, max)	−0.31 (−6.25, −1.68, 0.87, 2.44)	−0.32 (−4.75, −1.73, 0.41, 3.07)
< third percentile, *n* (%)	9 (20.5)	22 (21.6)
Last measurement *z* score, *n*	44	102
Mean (s.d.)	−2.02 (1.71)	−0.54 (1.51)
Median (min, Q1, Q3, max)	−2.02 (−6.62, −3.40, −0.93, 2.14)	−0.35 (−5.50, −1.62, 0.56, 2.91)
< third percentile, *n* (%)	24 (54.5)	20 (19.6)
Delta *z* score from first measurement		
Mean (s.d.)	−1.45 (1.89)	0.08 (0.53)
Median (min, Q1, Q3, max)	−1.85 (−5.08, −2.78, −0.32, 3.14)	0.06 (−1.96, −0.16, 0.28, 2.17)
t value (*P* value)	−5.09 (<0.0001)	1.54 (0.13)
Time in between measurements, years		
Mean (s.d.)	1.27 (0.60)	3.39 (3.21)
Median (min, Q1, Q3, max)	1.53 (0.17, 0.80, 1.81, 1.99)	2.32 (0.50, 1.48, 3.53, 15.95)

^a^Length, not height, was measured in patients <2 years of age.

HPP, hypophosphatasia; max, maximum; min, minimum; Q, quartile; s.d., standard deviation.

**Figure 4:**
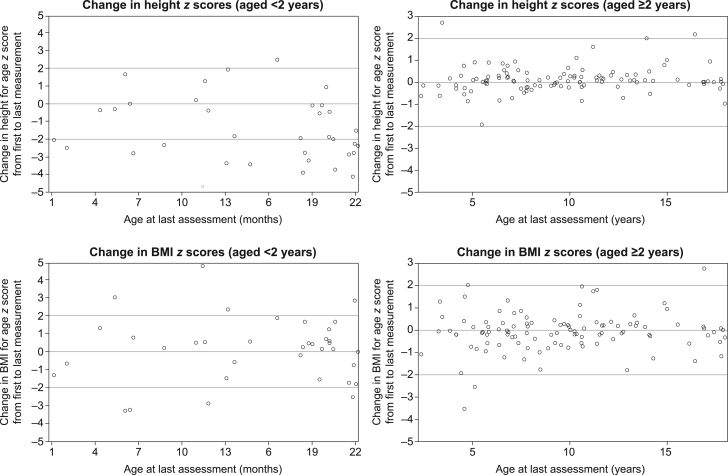
Change in height* and BMI *z* scores in children with HPP. *Length, not height, was measured in patients <2 years of age. BMI, body mass index; HPP, hypophosphatasia.

To test the robustness of these results, a sensitivity analysis was conducted in patients who had data from both aged <2 years and aged ≥2 years to assess growth throughout childhood (Fig. 4). Results of this analysis showed worsening in height (mean (s.d.) delta height *z* score: −0.86 (2.17); *n* = 20) and weight (delta weight *z* score: −0.81 (2.31); *n* = 23) over time, while BMI (delta BMI *z* score: 0.02 (1.74); *n* = 20) did not change for patients followed from <2 years onwards. In addition, because birth length may be less reliable than a length measurement obtained after birth, a second sensitivity analysis excluding birth length as a first measurement was conducted to assess the change from the first to last available measurement in children aged <2 years. Results of this analysis, while less pronounced, (mean (s.d.) delta length *z* score: −0.92 (1.42); *n* = 9) were largely consistent with the main results, indicating that the inclusion of birth length, which is prone to errors in measurement and artifacts resulting from rapid early infantile growth, did not influence the findings of the main analysis.

An analysis was also conducted to assess whether there was catch-up growth in the nine children aged <2 years whose height was below the third percentile at first assessment. This analysis showed an overall mean (s.d.) delta length *z* score of 0.88 (1.05) and that some children experienced substantial catch-up growth, with 4/9 (44.4%) of the children no longer having a height below the third percentile at the last recorded measurement. Among those aged ≥2 years (*n* = 22), spontaneous catch-up growth over time was limited (mean (s.d.) delta height *z* score: 0.28 (0.76)) with only 4/22 (18.2%) of the children no longer below the third percentile for height at last recorded measurement, indicating that many of the children who were short at first assessment remained so for the period assessed.

### Most children with HPP were found to be below their target height

An analysis based on the MPH of patients aged ≥2 years was conducted to understand whether children are on track to reach their genetic potential (MPH). Children with parental height data for both parents (*n* = 62) had a mean delta HT Z – MPH Z score of −0.66 (*P* value = 0.04); similar results were obtained whether one/both or no parents had a confirmed diagnosis of HPP (Table 4). Of note, 71.2% of those with available data had a delta HT Z – MPH Z score <0, while 42.4% had a delta HT Z – MPH Z score <−1. These findings indicate that, while most children with HPP had height *z* scores that were above the third percentile, they were substantially below the MPH.

**Table 4:** Target MPH *z* scores relative to parental HPP status and current height in children with HPP aged ≥2 years.

Variable	Total		Without parental HPP		With parental HPP	
	Height (cm)	*Z* score	Height (cm)	*Z* score	Height (cm)	*Z* score
Paternal height						
*n*	62	62	54	54	6	6
Mean (s.d.)	176.33 (8.28)	0.04 (1.16)	176.53 (7.91)	0.06 (1.11)	170.33 (5.79)	−0.80 (0.79)
Median (min, max)	175.65 (160.00, 198.00)	−0.07 (−2.21, 3.12)	176.50 (160.00, 193.04)	−0.05 (−2.21, 2.40)	171.50 (160.00, 177.00)	−0.65 (−2.21, 0.12)
***P***** value (comparing paternal *z* scores without parental HPP vs with parental HPP) = 0.0685**						
Maternal height						
n	62	62	45	45	15	15
Mean (s.d.)	163.21 (5.90)	0.01 (0.91)	163.94 (5.04)	0.13 (0.78)	161.24 (8.07)	−0.29 (1.25)
Median (min, max)	162.78 (145.00, 177.80)	−0.05 (−2.79, 2.27)	163.2 (150.70, 177.80)	0.01 (−1.92, 2.27)	160.00 (145.00, 172.72)	−0.48 (−2.79, 1.48)
***P***** value (comparing maternal *z* scores without parental HPP vs with parental HPP) = 0.2394**						
Target MPH						
*n*	62	62	42	42	18	18
Mean (s.d.)	169.14 (7.94)	0.03 (0.75)	169.88 (8.80)	0.08 (0.73)	167.45 (5.63)	−0.19 (0.70)
Median (min, max)	167.00 (154.40, 186.84)	0.04 (−2.01, 1.68)	169.40 (154.40, 186.84)	0.10 (−1.40, 1.51)	166.22 (159.87, 179.22)	−0.13 (−2.01, 0.87)
Delta HT Z – MPH Z						
*n*	n/a	62	n/a	42	n/a	18
Mean (s.d.)	n/a	−0.66 (1.31)	n/a	−0.63 (1.39)	n/a	−0.63 (1.17)
Median (min, max)	n/a	−0.61 (−4.74, 2.86)	n/a	−0.58 (−4.74, 2.86)	n/a	−0.59 (−2.77, 1.33)
t value (*P* value)		−2.27 (0.0363)		−2.93 (0.0056)		−3.96 (0.0002)

HPP, hypophosphatasia; HT Z, height *z* score; max, maximum; min, minimum; MPH, midparental height; MPH Z, midparental height *z* score; n/a, not applicable.

### Children with HPP were found to experience a broad range of manifestations regardless of height status

**Figure 5:**
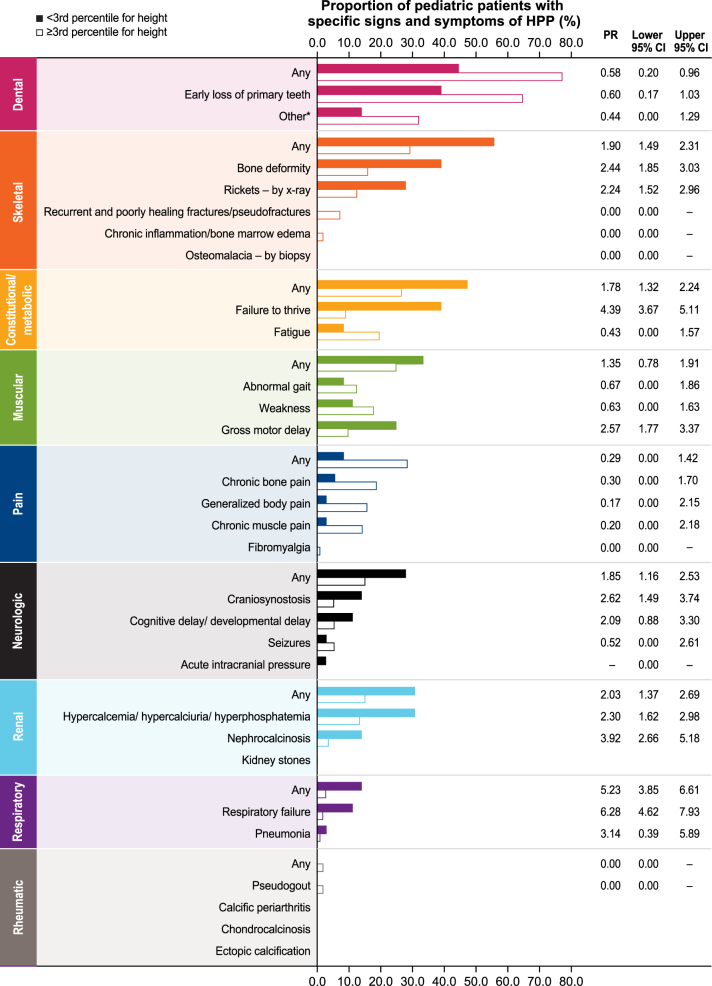
Frequency of specific signs and symptoms of HPP in children below (*n* = 36) and at or above (*n* = 113) the third percentile for height at last measurement. *Includes loss of permanent teeth, loose teeth, poor dentition, hypodontia, dental implants, dental bridges, and dentures. CI, confidence interval; HPP, hypophosphatasia; PR, prevalence ratio.

To determine whether the manifestations of HPP differ relative to height status, a comparison was done between children above and below the third percentile for height (*n* = 113 vs *n* = 36, respectively) at the last recorded assessment. Results of this analysis showed that similar signs and symptoms were experienced by children with short stature and those with normal stature, but the proportions varied. A higher proportion of children with short stature experienced skeletal (rickets and bone deformity), constitutional/metabolic (failure to thrive), muscular (gross motor delay), neurological, renal, and respiratory signs and symptoms of HPP when compared with children with normal stature (Fig. 5). Dental manifestations and pain were most prevalent in children with normal stature. Further investigation showed that 44/113 (38.9%) children above the third percentile for height had only dental manifestations compared with 5/36 (13.9%) children below the third percentile for height, suggesting that having odontohypophosphatasia only is not necessarily associated with normal stature. Moreover, children with normal stature experienced a similarly broad range of HPP manifestations as those below the third percentile for height, indicating substantial disease burden in some children despite not having short stature. Notably, the results of the analysis of delta height *z* score remained largely unchanged when those with odontohypophosphatasia were excluded (children aged <2 years: mean (s.d.) delta length *z* score = −1.44 (1.97); *n* = 40; children aged ≥2 years mean (s.d.) delta height *z* score = 0.06 (0.59); *n* = 70).

### Growth was impaired but stable in children who had their first HPP manifestation before 6 months of age

When examining age at first HPP manifestation in the <2-year age group, 46.2% (*n* = 18 out of 39 with data on onset age) were <6 months of age at first HPP manifestation (perinatal/infantile-onset HPP), and in the ≥2-year age group, 16.1% (*n* = 14 out of 87 with data on onset age) were <6 months of age at first HPP manifestation, and the remainder were 6 months of age or older at first HPP manifestation (juvenile-onset HPP). Thus, only 21 of the 94 patients with juvenile-onset HPP had data before the age of 2 years (Supplementary Table 2).

Regardless of whether the length/height measurement was taken before 2 years or after the age of 2 years, children with perinatal/infantile HPP were shorter for their age compared with those with juvenile-onset HPP. When comparing between perinatal/infantile-onset HPP and juvenile-onset HPP, mean height for age *z* scores were −1.11 vs −0.27, respectively, for those aged <2 years and −1.67 vs 0.46, respectively, for those aged ≥2 years. Notably, when evaluating change in height measurements in those ≥2 years of age, the mean delta height *z* score showed little change over time in both perinatal/infantile-onset HPP and juvenile-onset HPP cohorts (−0.14 and 0.14, respectively).

However, the change in height *z* score was more pronounced in patients with juvenile-onset HPP with data before 2 years of age compared with the perinatal/infantile-onset cohort (mean delta height *z* score: −1.83 vs −0.84). When specifically evaluating patients with perinatal/infantile onset, there was a slight decline in height *z* score before the age of 2 years (mean delta height *z* score = −0.84), while after the age of 2 years, height for age *z* score remained stable (mean delta height *z* score = −0.14). There was a greater decline in the height *z* score for children who were aged <2 years at the first assessment with perinatal/infantile-onset HPP compared with those with juvenile-onset HPP (−0.8 and −2.0, respectively).

Changes in weight and BMI *z* scores were also similar regardless of HPP onset in those aged ≥2 years (Supplementary Table 2). For those aged <2 years, changes in weight *z* scores remained similar regardless of age at first HPP manifestations, and BMI *z* scores improved in those with juvenile-onset HPP (Supplementary Table 2).

## Discussion

Short stature and failure to thrive have been previously described in children with HPP and were considered to be features of the disease (3, 4, 14, 15). Here, we report poor growth and weight gain during infancy (data collected before 2 years of age), although there was some catch-up growth potential in those who were below the third percentile for height. In data that were collected after 2 years of age, no change in height and weight trajectories was observed, indicating sustained longitudinal growth after 2 years of age.

While height *z* scores were within a normal range (above the third percentile) for most patients, the proportion of children with short stature was higher than expected from the population, and mean height *z* scores were significantly below the MPH (reduced by 0.66 s.d. compared to the patients’ target) regardless of whether the parent was affected by HPP. While more children with short stature experienced multiple manifestations of HPP than those without, many children (66.9%) in the total study population had two or more signs and symptoms of HPP.

When analyzing the change from the first to last available measurement, children aged <2 years had impaired growth and poor weight gain over time. As expected, BMI *z* scores did not change substantially. Two sensitivity analyses confirmed these findings in children aged <2 years but showed that there was considerable catch-up growth (gain in height *z* score 0.88 s.d.) among some children who were below the third percentile for height at first assessment. Conversely, among those aged ≥2 years who were below the third percentile for height at first assessment, catch-up growth was limited (only 18.2% of children were no longer below the third percentile for height at the last recorded measurement).

Collectively, the results of this study suggest that although poor growth may be observed in the first 2 years of life, poor growth is not a consistently observed characteristic of HPP in children assessed after 2 years of life. Specifically, those aged ≥2 years did not experience growth impairment whether the HPP symptoms presented before or after 6 months of age. This indicates that HPP might affect growth plate activity during the rapid period of growth during infancy (<6 months of age).

These results diverge somewhat from those of previously published reports, possibly due to differences in the patient populations. An analysis of data from 101 children attending a single center in the United States showed below median height from 2 to 20 years of age in both boys and girls with HPP (3). Although these patients exhibited steady growth, few experienced substantial spontaneous increases or decreases in height *z* scores over time, and only those with infantile-onset HPP had short stature (defined in the publication as height *z* score < −2). Weight was below average for all patients, but within the normal range for age and height (3).

This is the first study to assess anthropometric features of HPP using real-world data collected globally during routine clinical practice, which includes growth assessment relative to MPH ranges. Our results showed reduced height *z* scores of children relative to the MPH range (*P* value = 0.04) and reduced adult height of parents affected by HPP relative to parents without HPP status (*P* values did not reach statistical significance). Furthermore, this is the first study to investigate the signs and symptoms of HPP in children according to their height status. Although a higher proportion of children with normal stature had dental and pain manifestations compared with those having short stature, who were more likely to experience skeletal, constitutional/metabolic, muscular, neurologic, renal, and respiratory manifestations, both groups reported a high disease burden. These findings align with data from the Global HPP Registry which showed that adults with pediatric-onset HPP also exhibited a full spectrum of HPP manifestations (16). Future studies evaluating the signs and symptoms of childhood HPP can help characterize the real-world clinical profile of children not treated with asfotase alfa.

### Limitations

Given the observational nature of registry data collected from medical records during routine medical practice, there may have been variations in the availability of data due to differences in the standard of care from site to site and for different patients based on the severity of the disease. Limited data were available for patients with juvenile-onset HPP before the age of 2 years; results for this subgroup of patients should be interpreted with caution. For patients treated with asfotase alfa, only pretreatment data were included to ensure the inclusion only of natural history anthropometry. Growth data from untreated patients with perinatal HPP are basically unavailable in the registry since these children commence enzyme replacement therapy with asfotase alfa within days or weeks after birth. Also, patients were not posthumously enrolled on the registry. Therefore, it is unlikely that this analysis included severely affected patients who died early on before enrollment into the registry. Although it is possible that carriers or individuals with odontohypophosphatasia may have introduced bias, we stratified the change in height *z*-score by odontohypophosphatasia status, finding that the delta height *z*-score was similar when we excluded patients with odontohypophosphatasia. In addition, we were not able to predict final height since bone age was not recorded and no assumptions can be made on constitutional delay or pubertal growth. It is also unclear if, in general, bone age is reliable in patients with HPP who have impaired bone mineralization. No data regarding the progression of pubertal status throughout follow-up (or their eventual influence on growth) were available. Finally, the current inclusion criteria of the HPP Registry potentially allowed the entry of individuals without *ALPL* variants or simple carriers of *ALPL* variants. This registry limitation may have led to an underestimation of disease burden and short stature.

## Conclusions

Results of this study showed that height in children with HPP was significantly below the MPH target (−0.66 s.d.); however, short stature (< third percentile) was observed in a limited number of patients. Based on this, it can be speculated that the adult height of this study population might be somewhat below the population average, which is also supported by a median adult height *z* score between −0.48 and −0.65 for parents with HPP. Growth and, to a lesser extent, weight gain worsened over time in patients aged ≤2 years but not in patients aged ≥2 years, which is most probably related to a greater impact of the disease on the growth plates at a time when they are most active. Also, children with perinatal/infantile HPP were shorter for their age when compared with those who had juvenile-onset HPP. Lastly, skeletal signs and symptoms of HPP were more commonly observed among children with short stature compared with those who had normal stature; however, many children with HPP presented with two or more manifestations regardless of their anthropometric characteristics, suggesting that the disease burden may be independent of height status.

## Supplementary Material

Supplementary Material

## Declaration of interest

Anna Petryk and Shona Fang are employees of, and may own stock/options in, Alexion, AstraZeneca Rare Disease, Boston, MA, USA. Priya S Kishnani, Gabriel Ángel Martos-Moreno, Kathryn M Dahir, Agnès Linglart, Cheryl Rockman-Greenberg, Keiichi Ozono, Lothar Seefried, and Wolfgang Högler are consultants for, and have received research funding and honoraria from, Alexion, AstraZeneca Rare Disease, Boston, MA, USA.

## Funding

All aspects of this study were funded by Alexion, AstraZeneca http://dx.doi.org/10.13039/100004325 Rare Disease, Boston, MA, USA. Medical writing and editorial support were provided by Oxford PharmaGenesis Inc, Newtown, PA, USA, and funded by Alexion, AstraZeneca http://dx.doi.org/10.13039/100004325 Rare Disease, Boston, MA, USA.

## Availability of data and materials

Alexion, AstraZeneca Rare Disease will consider requests for disclosure of clinical study participant-level data provided that participant privacy is assured through methods such as data de-identification, pseudonymization, or anonymization (as required by applicable law), and if such disclosure was included in the relevant study informed-consent form or similar documentation. Qualified academic investigators may request participant-level clinical data and supporting documents (statistical analysis plan and protocol) pertaining to Alexion-sponsored studies. Further details regarding data availability and instructions for requesting information are available in the Alexion Clinical Trials Disclosure and Transparency Policy at http://alexion.com/our-research/research-and-development. Link to data-request form: https://alexion.com/contact-alexion/medical-information.
