# Supplementary material for: Growth and disease burden in children with hypophosphatasia
Source: Endocr Connect. 2023 Apr 25;12(5):e220240. doi: 10.1530/EC-22-0240 (PMC10160547; doi:10.1530/EC-22-0240)
Supplement: Supplementary Material [file supplementary_material.pdf]

Title: Growth and Disease Burden in Children With Hypophosphatasia

Wolfgang Högler, MD, FRCPCH[1,2], Agnès Linglart, MD, PhD[3], Anna Petryk, MD[4], Priya S. Kishnani, MD, MBBS[5], Lothar Seefried, MD[6], Shona Fang, MS, ScD[4], Cheryl Rockman-Greenberg, MD, CM, FRCPC, FCCMG[7], Keiichi Ozono, MD[8], Kathryn Dahir, MD[9], Gabriel Ángel Martos-Moreno, MD, PhD[10]

[1]Department of Paediatrics and Adolescent Medicine, Johannes Kepler University Linz, Linz, Austria; [2]Institute of Metabolism and Systems Research, University of Birmingham, Birmingham, UK; [3]AP-HP, Hôpital Bicêtre Paris Saclay, service d'endocrinologie et diabète de l'enfant, DMU 3 SEA, centre de référence des maladies rares du métabolisme du calcium et du phosphate, filière OSCAR; Université de Paris-Saclay INSERM U1185, Hôpital Bicêtre, Le Kremlin-Bicêtre, France; [4]Alexion, AstraZeneca Rare Disease, Boston, MA, USA; [5]Duke University Medical Center, Durham, NC, USA; [6]University of Würzburg, Würzburg, Germany; [7]University of Manitoba, Winnipeg, Manitoba, Canada; [8]Osaka University, Suita, Osaka, Japan; [9]Vanderbilt University Medical Center, Nashville, TN, USA; [10]Departments of Pediatrics and Pediatric Endocrinology Hospital Infantil Universitario Niño Jesús, IIS La Princesa, Universidad Autónoma de Madrid, CIBERobn, ISCIII, Madrid, Spain.

Corresponding author: Wolfgang Högler, MD, FRCPCH

Address: Department of Paediatrics and Adolescent Medicine, Johannes Kepler University Linz, Kepler University Hospital, Krankenhausstrasse 26-30, 4020 Linz, Austria

Phone/fax: +43 576808422001

Email: wolfgang.hoegler@kepleruniklinikum.at

OCRID iD: 0000-0003-4328-6304

Supplemental material

Plain language summary:

Hypophosphatasia (HPP) is a rare, inherited disease that can lead to bones not growing correctly. Children who have HPP may have problems with their growth, as measured by height and weight. This study was conducted to understand the relationships between factors, such as height and weight, and the symptoms of HPP in children.

The study used data from the Global HPP Registry, which is a database that collects information on patients with HPP. Patients enrolled in the Registry may be treated or not treated with enzyme replacement therapy (ERT). The group of Registry patients in this study includes both those patients who have been treated with ERT, and those patients who have never been treated with ERT. However, for those patients who have been treated with ERT, only data from prior to the start of ERT treatment were included in this study.

Results of the study showed that children with HPP tended to have poor growth before 2 years of age, as measured by both height and weight. However, their growth pattern was more normal between 2 and 18 years of age. The study also showed that when children with HPP reach adulthood, they may be slightly shorter than the height that they could achieve when compared with the height of their parents. Therefore, it is possible that the adult height of children with HPP may be somewhat below the normal adult height of the general population. The study also looked at the relationship between height and the symptoms of HPP in children. Both children with normal height and those who were shorter than normal for their age had many symptoms of HPP. However, there were differences between the two groups in the types of symptoms that each had, such as problems with bone development, muscles, breathing, and other parts of the body being more common in children who were shorter than normal for their age.

Supplemental Table 1: Changes in weight and BMI over time (based on at least 2 consecutive measurements per patient) in children with HPP

	**Children with HPP aged <2 years at assessment**	**Children with HPP aged ≥2 years at assessment**
**Weight**		
First measurement *z* score, n	49	101
Mean (SD)	-0.32 (1.31)	-0.33 (1.68)
Median (min, Q1, Q3, max)	-0.11 (-4.08, -1.23, 0.53, 2.14)	-0.11 (-5.62, -1.33, 0.79, 2.71)
<3rd percentile, n (%)	4 (8.2)	18 (17.8)
Last measurement *z* score, n	49	101
Mean (SD)	-0.99 (1.95)	-0.20 (1.57)
Median (min, Q1, Q3, max)	-1.10 (-3.97, -2.42, -0.40, 7.45)	0.01 (-5.78, -1.01, 0.71, 2.88)
<3rd percentile, n (%)	16 (32.7)	12 (11.9)
Delta weight *z* score from first measurement		
Mean (SD)	-0.68 (1.91)	0.13 (0.73)
Median (min, Q1, Q3, max)	-0.90 (-4.43, -1.79, 0.35, 7.00)	0.03 (-1.40, -0.25, 0.39, 4.28)
t value (*P* value)	-2.48 (0.02)	1.81 (0.07)
Time in between measurements, years		
Mean (SD)	1.39 (0.54)	3.41 (3.23)
Median (min, Q1, Q3, max)	1.59 (0.17, 0.99, 1.83, 1.99)	2.34 (0.50, 1.56, 3.53, 15.95)
**BMI**		

First measurement *z* score, n	44	101
Mean (SD)	-0.08 (1.57)	0.25 (1.11)
Median (min, Q1, Q3, max)	0.02 (-4.40, -0.99, 0.87, 3.35)	0.20 (-2.67, -0.41, 0.93, 3.22)
<3rd percentile, n (%)	5 (11.4)	3 (3.0)
Last measurement *z* score, n	44	101
Mean (SD)	0.18 (1.14)	0.24 (1.17)
Median (min, Q1, Q3, max)	0.24 (-2.37, -0.61, 1.03, 2.66)	0.36 (-4.42, -0.43, 0.99, 2.65)
<3rd percentile, n (%)	1 (2.3)	3 (3.0)
Delta *z* score from first measurement		
Mean (SD)	0.25 (1.65)	-0.02 (0.84)
Median (min, Q1, Q3, max)	0.46 (-3.26, -0.61, 1.14, 4.75)	-0.07 (-2.53, -0.40, 0.30, 2.77)
t value (*P* value)	1.02 (0.31)	-0.19 (0.85)
Time in between measurements, years		
Mean (SD)	1.27 (0.60)	3.40 (3.23)
Median (min, Q1, Q3, max)	1.53 (0.17, 0.80, 1.81, 1.99)	2.34 (0.50, 1.56, 3.53, 15.95)

BMI, body mass index; HPP, hypophosphatasia; max, maximum; min, minimum; Q, quartile; SD, standard deviation.

Supplemental Table 2: Changes in height,* weight, and BMI over time in children with HPP based on age at first HPP manifestation (first signs and symptoms < or ≥ 6 months)

	Children with HPP aged <2 years at assessment	Children with HPP aged ≥2 years at assessment
**Aged <6 months at first HPP manifestation**[†]		
First height measurement *z* score, n	18	14
Mean (SD)	-1.11 (2.32)	-1.67 (1.84)
Median (min, Q1, Q3, max)	-1.26 (-6.25, -2.58, 0.59, 2.17)	-1.84 (-4.75, -3.39, -0.58, 1.98)
Last height measurement *z* score, n	18	14
Mean (SD)	-1.95 (1.97)	-1.81 (1.94)
Median (min, Q1, Q3, max)	-1.92 (-6.62, -3.38, -0.89, 2.14)	-1.89 (-5.50, -2.97, -0.51, 2.21)
Delta height *z* score from first to last measurement, n	18	14
Mean (SD)	-0.84 (1.81)	-0.14 (0.50)
Median (min, Q1, Q3, max)	-0.80 (-3.37, -2.38, 0.02, 3.14)	-0.05 (-1.00, -0.56, 0.19, 0.89)
t value (*P* value)	-1.98 (0.06)	-1.03 (0.32)
First weight measurement *z* score, n	20	14
Mean (SD)	-0.17 (1.17)	-1.32 (1.98)
Median (min, Q1, Q3, max)	-0.06 (-1.95, -1.27, 0.52, 2.14)	-1.02 (-5.62, -2.10, -0.08, 1.62)
Last weight measurement *z* score, n	20	14
Mean (SD)	-0.85 (2.62)	-1.15 (2.07)
Median (min, Q1, Q3, max)	-1.08 (-3.97, -2.44, -0.11, 7.45)	-0.62 (-5.78, -1.75, -0.05, 1.41)

Delta weight z score from first to last measurement, n	20	14
Mean (SD)	-0.68 (2.33)	0.17 (0.47)
Median (min, Q1, Q3, max)	-1.31 (-3.30, -2.36, 0.31, 7.00)	0.16 (-0.66, -0.17, 0.56, 0.98)
t value (*P* value)	-1.32 (0.20)	1.31 (0.21)
First BMI measurement *z* score, n	18	14
Mean (SD)	0.30 (1.92)	0.01 (0.95)
Median (min, Q1, Q3, max)	0.34 (-4.40, -0.49, 1.56, 3.35)	0.13 (-1.80, -0.60, 0.77, 1.51)
Last BMI measurement *z* score, n	18	14
Mean (SD)	0.06 (1.32)	0.03 (1.20)
Median (min, Q1, Q3, max)	0.20 (-2.37, -1.00, 0.82, 2.54)	0.48 (-2.53, -0.98, 0.89, 1.19))
Delta BMI z score from first to last measurement, n	18	14
Mean (SD)	-0.23 (1.81)	0.02 (0.85)
Median (min, Q1, Q3, max)	0.11 (-3.26, -1.53, 0.79, 3.02)	0.16 (-2.53, -0.26, 0.34, 1.29)
t value (*P* value)	-0.55 (0.59)	0.09 (0.93)
**Aged 6 months to <18 years at first HPP manifestation**[‡]		
First height measurement *z* score, n	21	73
Mean (SD)	-0.27 (1.63)	-0.46 (1.47)
Median (min, Q1, Q3, max)	-0.20 (-3.64, -1.26, 0.98, 2.44)	-0.12 (-4.47, -1.58, 0.45, 3.07)
Last height measurement *z* score, n	21	73
Mean (SD)	-2.10 (1.63)	-0.32 (1.38)
Median (min, Q1, Q3, max)	-2.10 (-4.62, -3.42, -0.78, 1.23)	-0.10 (-3.60, -1.09, 0.57, 2.91)

Delta height z score from first to last measurement, n	21	73
Mean (SD)	-1.83 (1.92)	0.14 (0.56)
Median (min, Q1, Q3, max)	-2.00 (-5.08, -3.21, -0.92, 2.52)	0.08 (-1.96, -0.07, 0.31, 2.17)
t value (*P* value)	-4.37 (<0.001)	2.18 (0.03)
First weight measurement *z* score, n	22	72
Mean (SD)	-0.34 (1.31)	-0.15 (1.66)
Median (min, Q1, Q3, max)	-0.24 (-3.21, -1.23, 0.78, 1.62)	0.06 (-4.86, -1.17, 1.01, 2.71)
Last weight measurement *z* score, n	22	72
Mean (SD)	-1.28 (1.31)	-0.02 (1.49)
Median (min, Q1, Q3, max)	-1.18 (-3.57, -2.42, -0.55, 1.16)	0.25 (-4.01, -1.00, 0.85, 2.88)
Delta weight *z* score from first to last measurement, n	22	72
Mean (SD)	-0.94 (1.66)	0.13 (0.82)
Median (min, Q1, Q3, max)	-1.38 (-4.43, -2.04, 0.18, 2.35)	0.01 (-1.40, -0.28, 0.43, 4.28)
t value (*P* value)	-2.65 (0.02)	1.38 (0.17)
First BMI measurement *z* score, n	21	72
Mean (SD)	-0.31 (1.12)	0.35 (1.15)
Median (min, Q1, Q3, max)	-0.21 (-2.32, -1.01, 0.44, 1.95)	0.29 (-2.67, -0.38, 1.16, 3.22)
Last BMI measurement *z* score, n	21	72
Mean (SD)	0.10 (1.06)	0.31 (1.21)
Median (min, Q1, Q3, max)	0.03 (-1.58, -0.65, 0.93, 2.66)	0.37 (-4.42, -0.46, 1.09, 2.65)

Delta BMI *z* score from first to last measurement, n	21	72
Mean (SD)	0.41 (1.30)	-0.04 (0.91)
Median (min, Q1, Q3, max)	0.47 (-2.51, -0.32, 1.17, 2.84)	-0.10 (-1.92, -0.57, 0.29, 2.77)
t value (*P* value)	1.44 (0.17)	-0.34 (0.74)

*Length, not height, was measured in patients <2 years of age.

†Mean duration between first and last measurements was 1.3 years for those aged <2 years at assessment and 3.9 years for those aged >2 years at assessment.

‡Mean duration between first and last measurements was 1.4 years for height and BMI *z* scores and 1.5 years for weight *z* scores for those aged <2 years at assessment, and 3.6 years for all anthropometric measures for those aged >2 years at assessment.

BMI, body mass index; HPP, hypophosphatasia; max, maximum; min, minimum; Q, quartile; SD, standard deviation.

Supplemental Figure 1: First weight measurement in children with HPP aged <2 years

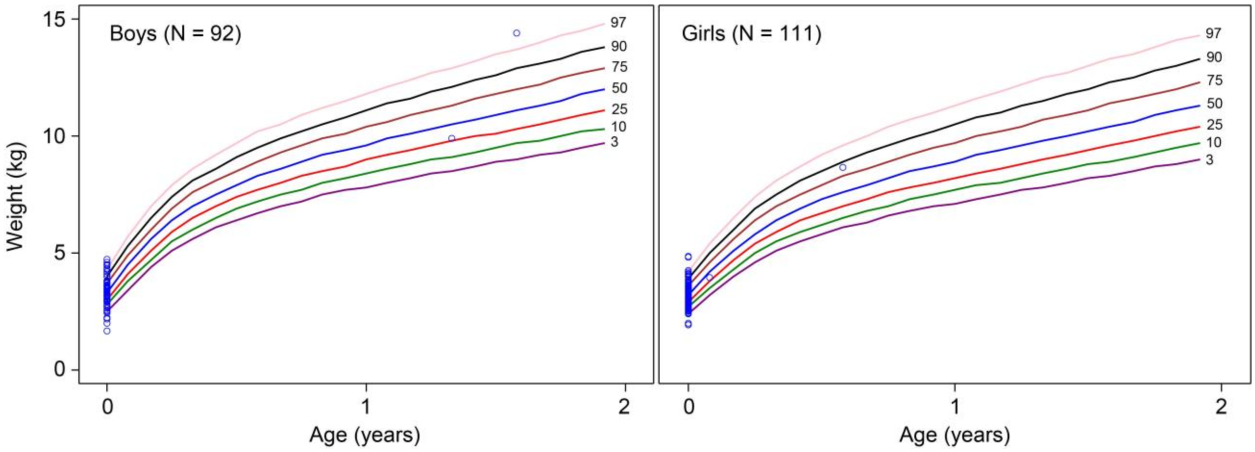

Weight measurements were analyzed in 203 children aged <2 years and are shown according to the WHO standards for weight.

HPP, hypophosphatasia; WHO, World Health Organization.

Supplemental Figure 2: First weight measurement in children with HPP aged ≥2 years

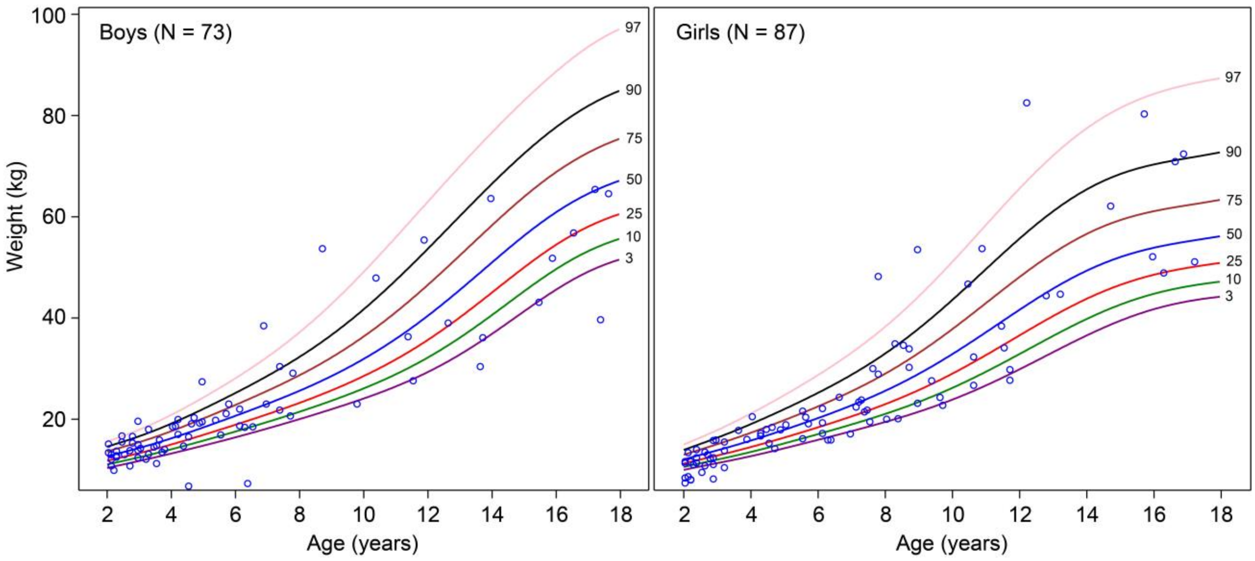

Weight measurements were analyzed in 160 children aged ≥2 years and are shown according to the CDC standards for weight.

CDC, Centers for Disease Control and Prevention; HPP, hypophosphatasia.

Supplemental Figure 3: First BMI measurement in children with HPP aged <2 years

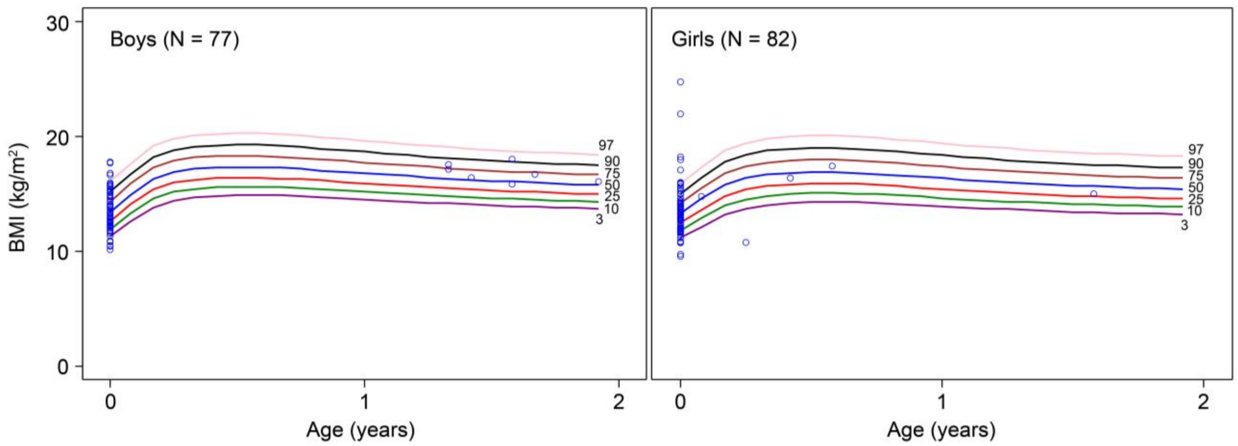

BMI measurements were analyzed in 159 children aged <2 years and are shown according to the WHO standards for BMI.

BMI, body mass index; HPP, hypophosphatasia; WHO, World Health Organization.

Supplemental Figure 4: First BMI measurement in children with HPP aged ≥2 years

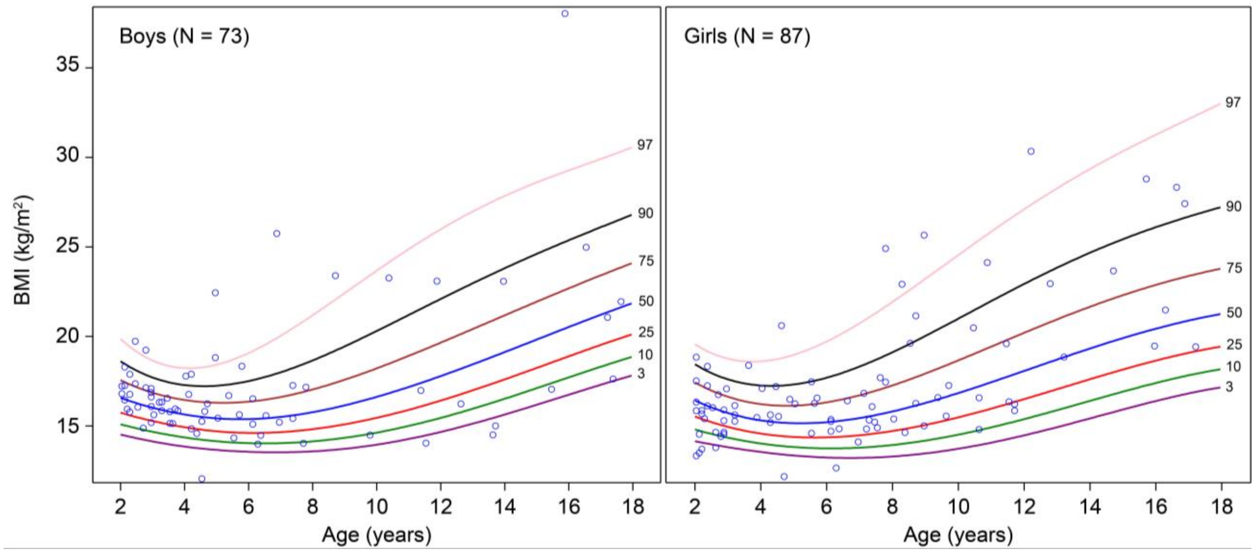

BMI measurements were analyzed in 160 children aged ≥2 years and are shown according to the CDC standards for BMI.

BMI, body mass index; CDC, Centers for Disease Control and Prevention; HPP, hypophosphatasia.
